# PD55 Living Recommendations Within A Type 2 Diabetes Mellitus Guideline

**DOI:** 10.1017/S0266462324003131

**Published:** 2025-01-07

**Authors:** Soledad Isern De Val, Patricia Gavin Benavent

## Abstract

**Introduction:**

The Institute for Health Sciences of Aragon (IACS) has coordinated the development of a clinical practice guideline (CPG), funded by the Spanish Ministry of Health, for managing antidiabetic drugs in patients with type 2 diabetes mellitus. We conducted a living systematic review to provide a continuously updated evidence summary for formulating living recommendations on intensifying basal insulin therapy by comparing glucagon-like peptide-1 (GLP-1) receptor agonists with rapid-acting insulin.

**Methods:**

In 2021, the IACS joined the Living Evidence to Inform Health Decisions (LE-IHD) project, which includes the use of technological tools to identify early emerging evidence on a defined topic. We conducted searches in the centralized Living Overview of Evidence (LOVE) and Epistemonikos databases. Specifically, the LOVE topic of interest from which the articles were selected was “glucagon-like peptide analogs and agonists for diabetes mellitus”. We applied the GRADE approach to rate the certainty of evidence and to develop clinical practice recommendations.

**Results:**

The initial baseline report included six randomized controlled trials (RCTs). We have continuously monitored the evidence and performed a monthly screening. Only one RCT was identified in the first update. The Guideline Development Group (GDG) decided to formulate a strong recommendation in favor of GLP-1 receptor agonists. The GDG considered the reduction in the risk of severe hypoglycemia events among patients treated with GLP-1 receptor agonists, compared with rapid insulin, particularly significant as well as the greater improvement in patient quality of life. Since the guideline was formulated, no new evidence has been identified that would change the recommendation.

**Conclusions:**

Adoption of the living systematic approach underscores the commitment to providing continuously updated recommendations within CPGs. Utilizing the GRADE approach, the Guideline Development Group decided to formulate a strong recommendation in favor of GLP-1 receptor agonists over rapid insulin. No new evidence has emerged to alter this recommendation. The living systematic review will remain active, ensuring continuous monitoring until the final draft CPG is disseminated.

